# Supplementary material for: Dynamic Expression of Imprinted Genes in the Developing and Postnatal Pituitary Gland
Source: Genes (Basel). 2021 Mar 30;12(4):509. doi: 10.3390/genes12040509 (PMC8066104; doi:10.3390/genes12040509)
Supplement: Supplementary file 1 [file genes-12-00509-s001.zip › Supplementary/Suppl-Figures-proofreading.docx]

Supplementary Figures


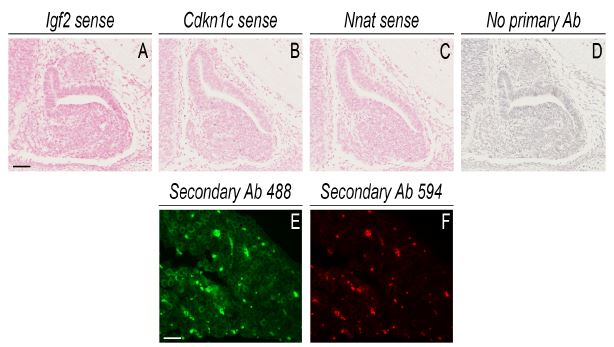


**Figure S1.** Negative controls for mRNA *in situ* hybridisation (ISH), immunohistochemistry (IHC) and immunofluorescence (IF). ISH performed using *Igf2* (**A**), *Cdkn1c* (**B**) and *Nnat* (**C**) sense probes and IHC performed using goat α-rabbit secondary antibody biotinylated in absence of primary antibody (**D**), which serves as negative control for both GRB10 and SOX2 in Figure 2. E and F show sections stained with goat α-mouse secondary antibody conjugated with a 488-fluorochrome (**E**) or goat α-rabbit secondary antibody conjugated with a 594-fluorochrome (**F**) in absence of primary antibody, which represent the negative control for DDC and GH, respectively, in the IF experiment shown in Figure 4. Scale bar in A represents 50 μm for A-D; scale bar in E represents 25 μm for E-F (*n* = 1).


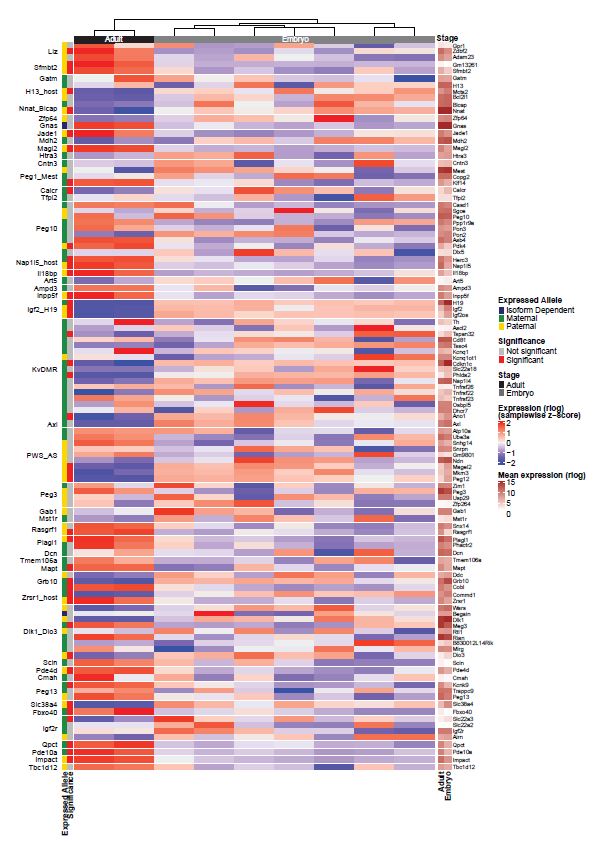


**Figure S2.** Heatmap showing expression of imprinted genes in anterior pituitary (AP) samples in embryo and adult. Data from individuals shown in Figure 1. (A) Expressed imprinted genes (right-sided labels) are sorted by chromosomal coordinate and each block corresponds to either a known imprinted gene cluster or a single imprinted gene (left-sided labels). Each imprinted gene is either paternally expressed, maternally expressed, or its expressed allele is isoform-dependent. Statistical significance for differential expression between embryo and adult was determined based on a P-value (corrected for multiple hypothesis testing based on the Benjamini-Hochberg procedure) threshold of 0.05 and a fold change threshold of 4. A regularized log transformation was applied to normalized counts (see Materials and Methods), and the values were then z-transformed across samples. Mean expression values in adult and embryo samples are represented in the last two columns.


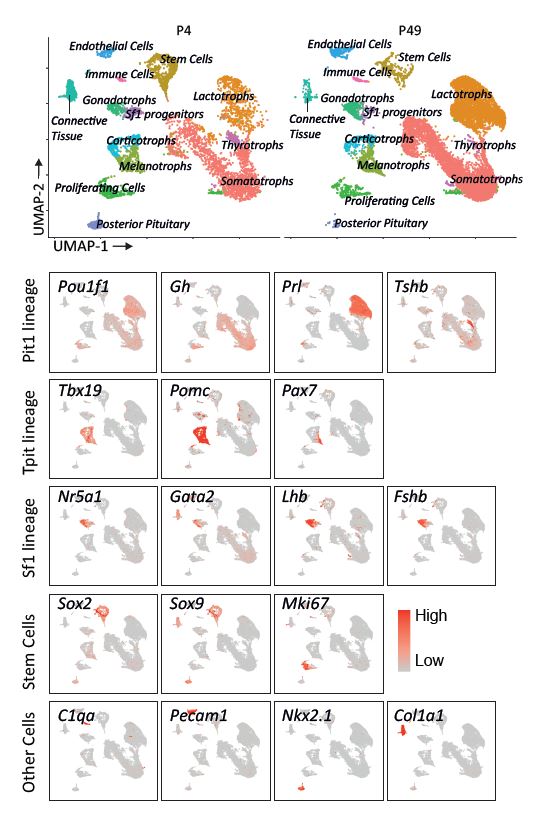


**Figure S3.** Identification of pituitary cell types in postnatal anterior pituitary (AP) scRNAseq. Expression of known endocrine cell markers are detected and enriched clusters are named accordingly. *Gh* (Somatotrophs), *Prl* (Lactotrophs) and *Tshb* (Thyrotrophs) all demarcate the Pit1 (*Pou1f1)* lineage. Cells expressing *Pomc* (Corticotrophs), *Pomc+Pax7+* (Melanotrophs) are housed within the Tpit (*Tbx19)* lineage. *Gata2, Lhb* and *Fshb* are all present in the Gonadotrophs while lower expression of these committed markers is seen Sf1 (*Nr5a1*) progenitors. Stem cells are enriched with *Sox2* and *Sox9,* whilst *Mki67* highlights the Proliferating Cells. Non-endocrine cells are also present in the datasets; *C1qa* (Immune Cells), *Pecam1* (Endothelial Cells), *Nkx2.1* (Posterior Pituitary) and *Col1a1* (Connective Tissue). Grey to red indicates no expression to high expression.


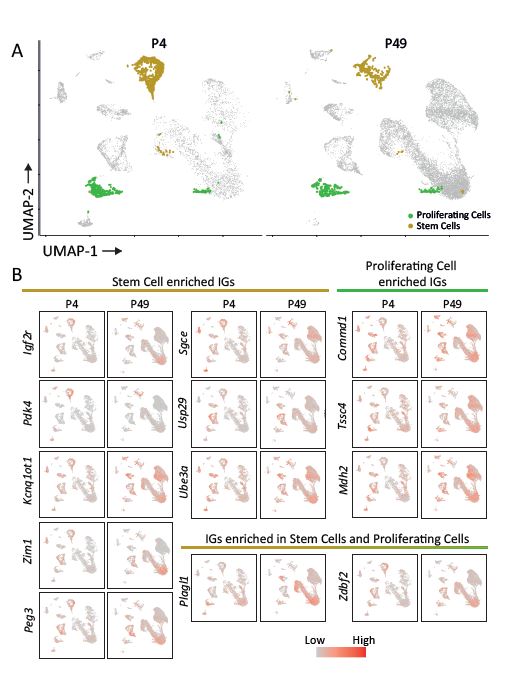


**Figure S4.** Single cell analysis of stem cell enriched imprinted gene expression in postnatal AP. UMAP projection of P4 and P49 cells with ‘Proliferating Cells’ and ‘Stem Cells’ clusters highlighted. **B**) IGs found enriched specifically in the Stem Cell population and Proliferating Cell population (Table 2) are plotted for each age as well as the IGs (*Plagl1* and *Zdbf2*) that are found significantly upregulated in both these clusters. Grey to red indicates no expression to high expression.


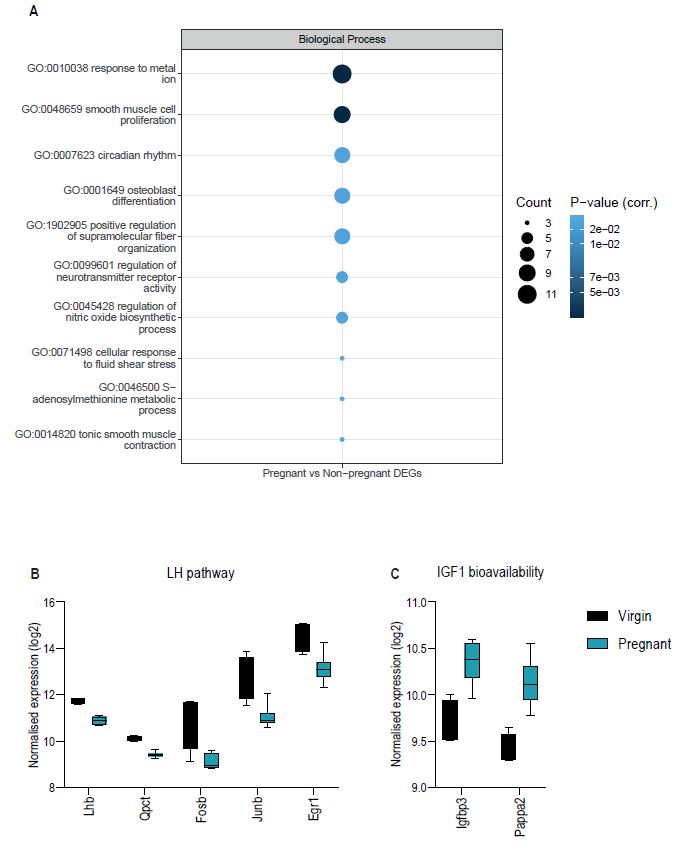


**Figure S5.** Differentially expressed (DE) genes comparing virgin and pregnant pituitary gland. (**A**) Enrichment in Gene Ontology (GO) biological process terms for differentially expressed genes. Significant GO terms were defined at FDR <0.05. (B and C) DE genes in the luteinising hormone (LH) pathway (**B**) and IGF1 bioavailability pathway from the transcriptomics data shown in Figure 4C. Box and whiskers plots show mean and min-max range of the data.


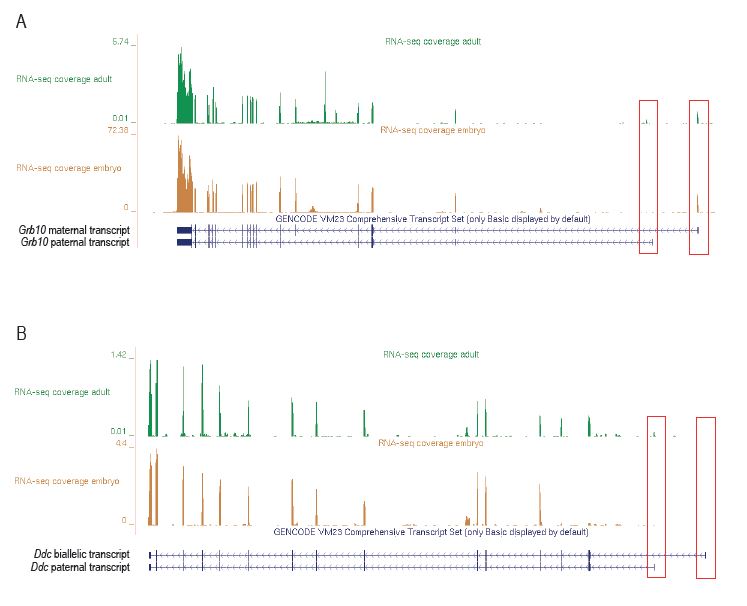


**Figure S6.** Genome browser view of sequenced reads aligned to the *Grb10* and *Ddc* genomic regions. University of California Santa Cruz (UCSC) Genomics Institute Genome Browser view of RNAseq data from embryonic and adult pituitaries described in Figure 1. The *Grb10* and *Ddc* loci in mouse have annotated transcripts which utilise alternative transcriptional starts (red boxes). (**A**) At the *Grb10* locus there are RNAseq reads that map to both the maternal and paternal transcripts in adult, and predominantly the maternal transcript in the embryo. (**B**) At the *Ddc* locus transcription in the adult appear to initiate from the paternal-specific promoter region. Embryonic transcripts appear to initiate at exon 4.
